# Correction of UAV LiDAR-derived grassland canopy height based on scan angle

**DOI:** 10.3389/fpls.2023.1108109

**Published:** 2023-03-20

**Authors:** Cong Xu, Dan Zhao, Zhaoju Zheng, Ping Zhao, Junhua Chen, Xiuwen Li, Xueming Zhao, Yujin Zhao, Wenjun Liu, Bingfang Wu, Yuan Zeng

**Affiliations:** ^1^ State Key Laboratory of Remote Sensing Science, Aerospace Information Research Institute, Chinese Academy of Sciences, Beijing, China; ^2^ University of Chinese Academy of Sciences, Beijing, China; ^3^ State Key Laboratory of Vegetation and Environmental Change, Institute of Botany, Chinese Academy of Sciences, Beijing, China; ^4^ School of Ecology and Environmental Science, Yunnan University, Kunming, Yunnan, China

**Keywords:** UAV lidar, grassland, canopy height, scan angle, height loss

## Abstract

Grassland canopy height is a crucial trait for indicating functional diversity or monitoring species diversity. Compared with traditional field sampling, light detection and ranging (LiDAR) provides new technology for mapping the regional grassland canopy height in a time-saving and cost-effective way. However, the grassland canopy height based on unmanned aerial vehicle (UAV) LiDAR is usually underestimated with height information loss due to the complex structure of grassland and the relatively small size of individual plants. We developed canopy height correction methods based on scan angle to improve the accuracy of height estimation by compensating the loss of grassland height. Our method established the relationships between scan angle and two height loss indicators (height loss and height loss ratio) using the ground-measured canopy height of sample plots with 1×1m and LiDAR-derived heigh. We found that the height loss ratio considering the plant own height had a better performance (R^2^ = 0.71). We further compared the relationships between scan angle and height loss ratio according to holistic (25–65cm) and segmented (25–40cm, 40–50cm and 50–65cm) height ranges, and applied to correct the estimated grassland canopy height, respectively. Our results showed that the accuracy of grassland height estimation based on UAV LiDAR was significantly improved with R^2^ from 0.23 to 0.68 for holistic correction and from 0.23 to 0.82 for segmented correction. We highlight the importance of considering the effects of scan angle in LiDAR data preprocessing for estimating grassland canopy height with high accuracy, which also help for monitoring height-related grassland structural and functional parameters by remote sensing.

## 1 Introduction

Grassland canopy height is an essential structural trait, which is usually regarded as one of the key indicators for representing grassland functional diversity (Fisher et al., 2018; Rossi et al., 2020; White et al., 2022). It is also used to classify grassland types (Dixon et al., 2014; Wu et al., 2017), and further to characterize various ecosystem functions, such as aboveground biomass (Fassnacht et al., 2021), species diversity (Sankey et al., 2021; Gholizadeh et al., 2022b) and grazing intensity (Bi et al., 2018). The estimation of grassland canopy height can facilitate grassland ecosystem monitoring and adaptive management.

Given the importance of grassland canopy height to grassland ecosystems, it is particularly crucial to achieving regional grassland canopy height mapping with high accuracy. Compared with ground data surveys at the sample scale, remote sensing provides spatially continuous data for canopy height estimation over a large region. For optical remote sensing data, some studies estimated the grassland canopy height successfully based on random forest models, but numerous parameters (i.e., vegetation indices, climate and topographic factors) were needed as inputs and they were often inconsistent across grassland types (Spagnuolo et al., 2020; Yin et al., 2020; Dusseux et al., 2022). Moreover, since the vegetation indices are highly susceptible to change due to the effects of other factors such as view angle, canopy structure and topography (Sims et al., 2011; Chen et al., 2020; Gu et al., 2021), it is difficult to find a universal vegetation index to monitor grassland canopy height. A previous study even failed to find the relationships between multiple vegetation indices (VIs) and grassland canopy height (Tiscornia et al., 2019). In addition, UAV-borne ultrahigh-resolution imagery are also used for the extraction of canopy height or other structural information based on structure-from-motion (SfM) photogrammetry methods, which can obtain point clouds from multiple images (Kalacska et al., 2017; Coops et al., 2021). But, the derived canopy height model (CHM) based on the SfM method shows some uncertainties in the vertical direction, especially for low plants (Cunliffe et al., 2016; Wijesingha et al., 2019), which influences the accuracy of grassland canopy height estimation (Michez et al., 2019; Zhang et al., 2022).

Light detection and ranging (LiDAR) offers new insight and technology for direct vegetation canopy height acquisition due to more powerful penetration ability than optical remote sensing techniques (Lefsky et al., 2002). LiDAR can provide both 3D spatial point cloud data and backscattered data (intensity data) for each observation point through active laser emission and reception as well as distinguish ground and non-ground points by multiple pulse-echo data (Bakx et al., 2019; Calders et al., 2020; Coops et al., 2021). Moreover, LiDAR point clouds allow us to get enough information without being limited by the spatial resolution of pixels (Jansen et al., 2019; Zheng et al., 2022). It has been widely used in obtaining the structure of forest ecosystems (Wulder et al., 2012; Zhao et al., 2018; Coops et al., 2021), and even global forest canopy height products based on space-borne LiDAR have been produced (Simard et al., 2011; Simard et al., 2018; Lang et al., 2022). In comparison, the application of LiDAR technology in grassland ecosystems is still finite due to the complexity of grassland (low height and high density) and the small size of individual plants. Although grassland canopy height can be estimated accurately based on terrestrial laser scanning (TLS) data (Guimarães-Steinicke et al., 2019; Tian et al., 2020; Xu et al., 2020), it is limited to the sample plot scale. On the contrary, space-borne LiDAR is too coarse to detect grassland canopy height because of low point density or large laser footprint, even if it can cover large regional or global areas (Malambo and Popescu, 2021). While providing relatively high point density data, UAV LiDAR offers the possibility to scale from sample plot to region for monitoring the canopy height of grasslands directly (Da Costa et al., 2021; Zhang et al., 2021), which has been successfully used to monitor the canopy height of forests, shrubs, and crops (Zalite et al., 2016; Liu et al., 2018b; Zhao et al., 2021).

Several studies, which explored the potential of UAV LiDAR to estimate canopy height in grasslands, reported height underestimation (Miura et al., 2019; Zhao et al., 2022). The underestimation would affect the estimation of grassland ecosystem functions related to canopy height and its heterogeneity. For example, Da Costa et al. (2021) found that grassland aboveground biomass estimated based on UAV LiDAR-derived canopy height was lower than that measured on the ground. High-density canopy was considered to be a main cause in forests, which hamper the penetration of laser pulses to reach the ground (Hu et al., 2020). Although grasslands may have a higher canopy density than forests, the gaps between grass individuals combined with high point cloud density allow UAV LiDAR to obtain reliable ground elevation information (Getzin et al., 2021; Zhao et al., 2022). The height loss at canopy was proved to be the main cause which greatly reduced the accuracy of grassland height-related structural traits (Miura et al., 2019). Zhao et al. (2022) investigated the height information loss distributed in grassland canopy top and bottom by comparing UAV LiDAR data with TLS data and ground-measured data. However, there are few studies exploring the differences of canopy height loss in the horizontal direction perpendicular to flight routes and the influence factors contributing to this discrepancy. Scan angle proves to be a factor severely affecting the prediction of structural traits in forests (Keränen et al., 2016; Liu et al., 2018a; Dayal et al., 2022) and narrower scan angle usually leads to a more accurate estimation of the mean height. But now, the effects of scan angle on estimating mean height of grassland are still unclear, which is essential for developing robust LiDAR-based models for regional grassland height estimation with various types.

In this study, we aim to estimate the grassland canopy height based on UAV LiDAR data and further quantify the impacts of scan angle on the loss of canopy height estimation towards a temperate meadow steppe. The research questions of this study include (1) how much uncertainty there is in estimating grassland height with UAV LiDAR data; (2) how much does the loss of grassland height relate to the scan angle, especially for the different height layers of grassland and (3) how much can the accuracy of the UAV LiDAR-derived grassland height be improved after correction based on scan angle. We explore these questions by establishing the relationships between the height loss indicators and scan angle, which are further used to correct grassland canopy height, and comparing with *in situ* data to demonstrate the feasibility of the correction method. Our study highlights that the accuracy of UAV LiDAR-derived grassland canopy height estimation can be improved based on scan angle, providing a reference to map grassland canopy height and height-related parameters more accurately at regional scale.

## 2 Materials and methods

### 2.1 Study area

This study is conducted at the National Hulunbuir Grassland Ecosystem Observation and Research Station (NHGEORS) (central coordinates: 49°19ˊ56″N, 119°57ˊ18″E) located in the center of the Hulunbuir meadow steppe in the northeast of Inner Mongolia, China (**Figure 1**). It is one of the most typical temperate meadow steppes in China, belonging to the temperate sub-humid zone with mean annual precipitation of 380–400mm, mean annual temperature of -2–1°C and humidity of 49%–50% (Barneze et al., 2022; Shen et al., 2022). The landform is the undulating hill in the piedmont of Greater Khingan Mountains with the elevation ranging from 650m to 700m. The main soil is the light loamy chernozem and dark chestnut soil developed on the parent material of loess.

**Figure 1:**
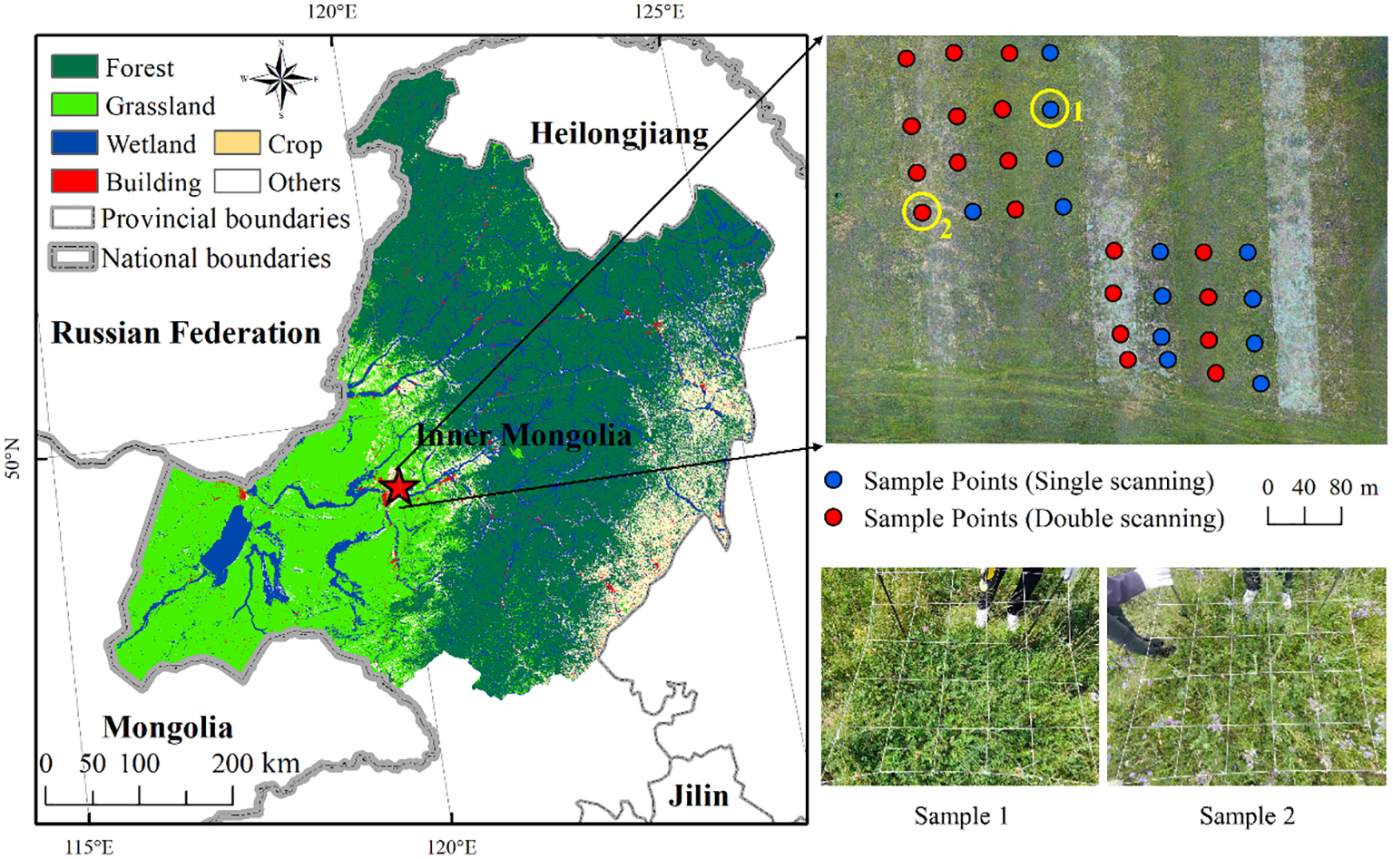
The location of National Hulunbuir Grassland Ecosystem Observation and Research Station (red star) and land cover data with 10m spatial resolution from ChinaCover2020 (left) (Wu et al., 2017), and UAV RGB image with 32 field-measured sample plots and photographs (right).

As the typical area for observation and research on the structure and function of temperate meadow grassland ecosystem, more than 30 dominant species were recorded in our field measurements and several grassland communities could be distinguished, including Poaceae (*Leymus chinensis, Stipa baicalensis* and *Cleistogenes squarrosa*), Fabaceae (*Astragalus laxmannii* and *Oxytropis myriophylla*), Asteraceae (*Artemisia scoparia*, *Artemisia frigida* and *Klasea centauroides*), Amaryllidaceae (*Allium tenuissimum* and *Allium polyrhizum*), and Ranunculaceae (*Thalictrum squarrosum* and *Clematis hexapetala*) (Zhu et al., 2019). The grassland structure in this region has obvious hierarchical differences, especially in the canopy height due to the high diversity of grassland species. A representative grassland area covering about 400×400m with rich species and significant canopy height differences is selected for UAV flight experiment and method development in this region.

### 2.2 Data acquisition

#### 2.2.1 Field measurements

The field measurements data were collected from August 1 to 6 in 2021. A total of 32 sample plots were uniformly distributed in the northwest and southeast regions of the study area. Each plot was 1×1m in size and it was divided into 25 investigation units with 0.2×0.2m. Within each unit, all species and their respective numbers were recorded and the height of each dominant species was measured at least three times from three different individual plants. Therefore, the mean height of each unit could be calculated as the mean height of each species, weighted by species abundance. Plot-level canopy height was obtained by averaging the mean height of each unit. Besides, the central coordinate pairs of each sample plot were recorded by Trimble GeoXH 3000 handheld GPS (Trimble Navigation Ltd, Sunnyvale, USA) and the differential correction was performed to minimize the position errors and obtain a decimeter-level positioning accuracy.

#### 2.2.2 UAV LiDAR data acquisition and preprocessing

The flight campaign for acquiring the UAV LiDAR data of the study area was conducted on August 6, 2021 using DJI M600 UAV platform (DJI, Shenzhen, China). LiAir VH Pro LiDAR scanning system (Green Valley Inc., Beijing, China) was equipped, emitting 905nm pulses at a frequency of 480,000 pulses/second with a detection range of about 320m. The integrated LiAir VH Pro system equipped with global navigation satellites system (GNSS) and inertial navigation system (INS) provided high-precision positioning information of 5cm for UAV LiDAR data. The UAV flew around 100–120 m above ground level with a flight speed of 5–6 m/s. The maximum scan angle of ±40° and more than 50% flight strip overlap were set, resulting in an average point cloud density of 248 points/m^2^. Additionally, UAV RGB images of the flight region with 2cm spatial resolution were obtained synchronously with LiDAR data acquisition, which could be conducive to determining the boundaries of sample plots. Among the ground sample plots, 19 sample plots were scanned twice with different scan angles by UAV laser scanner, while 13 sample plots were scanned once, thus, a total of 51 observation samples were acquired.

Before extracting the height of grassland canopy, preprocessing of denoising and filtering for UAV LiDAR data were performed. Outliers were identified and eliminated when the distance from the center point to its nearest neighboring point was more than a threshold, which was determined as five times standard deviation of the distance (mean distance + 5×std) in this study. After denoising, a local minimum filtering algorithm was applied to identify seed ground points within each investigation unit (0.2×0.2m), which was proved to be an effective method for ground finding (Wang et al., 2017; Zhao et al., 2022). The rest laser returns points were considered vegetation points. The vegetation and ground points were classified by the commercial software Terrasolid (Terrasolid, Helsinki, Finland).

### 2.3 Methods

#### 2.3.1 Estimation of mean canopy height and extraction of scan angle

There are several methods commonly used to extract height parameters, such as based on 95th or other percentiles (Zhao et al., 2022) and rasterized canopy height model (CHM) (Wang et al., 2017; Zhang et al., 2021). In this study, we estimated grassland canopy height by building the CHM. A digital surface model (DSM) and digital terrain model (DTM) with 0.1m spatial resolution were generated by triangulated irregular network interpolation based on vegetation points and ground points, respectively. CHM was established by subtracting DTM from DSM, and the value of each pixel was the estimated mean height of all plants within this pixel. To ensure the comparability with measured heights from ground sample plot (1×1m), we extracted all the pixels (10×10) within each ground sample plot from the CHM, which were located by central coordinates and high-precision UAV RGB images, and the mean CHM value of all pixels was calculated as the estimated mean grassland height by LiDAR. Meanwhile, the mean scan angle (absolute value) of all point clouds within these pixels at one scan was calculated as the scan angle of the corresponding observation sample.

#### 2.3.2 Division of modeling and validation datasets

We divided the sample plots into two datasets for the modeling and validation of height correction, respectively. The modeling dataset included the sample plots with single scanning and the first scan data of the sample plots with double scanning (N = 32), while the second scan data of the sample plots with double scanning were used as validation dataset (N = 19) (**Table 1**). From the table, similar data distributions were represented between two datasets and informed a reasonable division of our samples.

**Table 1:** Statistics of field-measured grassland canopy height and the LiDAR scan angle of the observation samples for modeling and validation datasets.

Dataset	Parameter	Maximum	Minimum	Mean	STD
Modeling dataset (N=32)	Height from ground (cm)	63.9	29.6	45.6	8.19
	Scan angle (°)	40	1	15.41	10.68
Validation dataset (N=19)	Height from ground (cm)	63.9	29.6	46.62	9.23
	Scan angle (°)	40	4	18.5	8.58

#### 2.3.3 Height correction

Two indicators were adopted to represent the height information loss in our study, including height loss (H_loss_) and height loss ratio (H_loss_ Ratio) calculated as follows:

$H_{loss}=H_{measured}-H_{estimated}$


$$H_{loss}\;Ratio=\;\frac{H_{loss}}{H_{measured}}$$

Where $H_{loss}$ was the loss of grassland canopy height at the level of sample plot; $H_{measured}$ and $H_{estimated}$ were the canopy height measured on the ground for each sample plot and their corresponding estimated mean canopy height based on UAV LiDAR data, respectively. $H_{loss}\;Ratio$ was the proportion of height information loss to the measured grassland canopy height.

The relationships between height loss indicators and scan angle were determined based on a simple linear regression model to quantify the effects of scan angle on grassland canopy height estimation. F-test was adopted to test the significance of these relationships at levels of 0.01 and 0.05. In order to further analyze the effect of plant height on the relationship between scan angle and the loss of grassland height, we also compared the performance of the relationships among three segmented height ranges of ground-measured canopy height (25–40cm with 12 samples, 40–50cm with 10 samples and 50–65cm with 10 samples), which was divided according to the plant functional groups with different plant competitive ability (Koyanagi et al., 2013; Ladouceur et al., 2019) as well as combined the common principle of sample equalization. Additionally, we used a t-test to test the significance of the differences in height loss between the three sets.

According to the determined relationships between height loss indicators and scan angle of the modeling dataset within holistic and segmented height ranges, two forms of correction method (holistic correction and segmented correction) were adapted to calculate two height loss indicators of the validation dataset and subsequently used them for the height loss correction as follows:


$$H_{estimated}^{'}=H_{estimated}+H_{loss}$$

$$H_{estimated}^{'}=H_{estimated}*\left(\frac{1}{1-H_{loss}\;Ratio}\right)$$

Where $H_{estimated}^{'}$ was the final grassland canopy height of each sample plot within the validation dataset estimated by UAV LiDAR data after correcting the loss of grassland height determined by $H_{loss}$ or $H_{loss}\;Ratio$ , which were obtained from the relationships between scan angle and them.

#### 2.3.4 Accuracy assessment before and after correction

We used the validation dataset to assess the performance of the height correction method by comparing the coefficient of determination (R^2^), root mean squared error (RMSE) and mean absolute percentage error (MAPE) before and after correction. Besides, the 1:1 line was used to measure the deviation of the UAV LiDAR-estimated grassland height from the ground-measured height. Detailed descriptions of the Eqs. were shown as follows:


$$R^{2}=1-\frac{\sum_{i=1}^{n}{\left(y_{i}-\hat{y_{i}}\right)}^{2}}{\sum_{i=1}^{n}{\left(y_{i}-\bar{y_{i}}\right)}^{2}}$$

$$RMSE=\sqrt{\frac{1}{n}\sum_{i=1}^{n}{\left(y_{i}-\hat{y_{i}}\right)}^{2}}$$

$$MAPE=\sum_{i=1}^{n}\left|\frac{y_{i}-\hat{y_{i}}}{y_{i}}\right|\times \frac{100\%}{n}$$

where n was the number of validation sample plots; $y_{i}$ and $\hat{y_{i}}$ were the measured and predicted values of the i^th^ sample plot; $\bar{y_{i}}$ was the mean measured value of all the validation sample plots.

## 3 Results

### 3.1 Grassland height estimation before correction

Grassland height estimated by UAV LiDAR showed a low accuracy (R^2^ = 0.29, *p*< 0.01, RMSE = 27.59cm) and a significant underestimation by comparing with the field-measured height (**Figure 2A**). The field-measured grassland canopy height for the plots of the modeling dataset distributed between 26.9cm and 63.9cm with an average of 45.6cm, while the corresponding UAV LiDAR-estimated height data only ranged from 10.5cm to 34.4cm with a mean height of 19.9cm.

**Figure 2:**
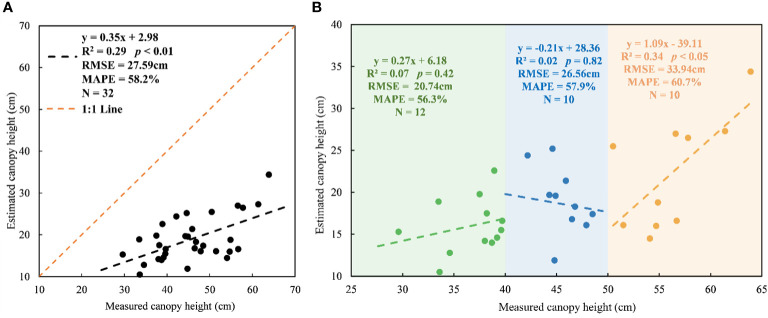
Plot-level measured canopy height versus estimated canopy height from CHM in whole height ranges **(A)** and three height ranges (25–40cm, 40–50cm, 50–65cm) **(B)** for the modeling dataset.

Our results also showed inconsistent performance of UAV LiDAR-derived grassland canopy height in three different height ranges (**Figure 2B**). Highest correlation between measured height and estimated height was found in the height range of 50–65cm (R^2^ = 0.34, *p*< 0.05), which better than that of all samples (**Figure 2A**), but it also had maximum underestimated deviations with MAPE of 60.7%. Beyond our expectations, UAV LiDAR basically failed to estimate the grassland height which was in the height range of 25–40cm and 40–50cm with R^2^ = 0.07 and 0.02 (*p* > 0.05) and even it showed a negative performance in the height range 40–50cm.

### 3.2 Relationships between scan angle and height loss

We compared the relationships between scan angle and two height loss indicators based on the samples of the modeling dataset shown in **Figure 3**. There was a stronger significant negative relationship between scan angle and height loss ratio (R^2^ = 0.71, *p*< 0.01) than height loss (R^2^ = 0.18, *p*< 0.01). In the scan angle range of 0–40°, the loss of grassland height estimated by UAV LiDAR ranged from 14.3cm to 40.1cm, while the ratio of the loss ranged from 42% to 74%.

**Figure 3:**
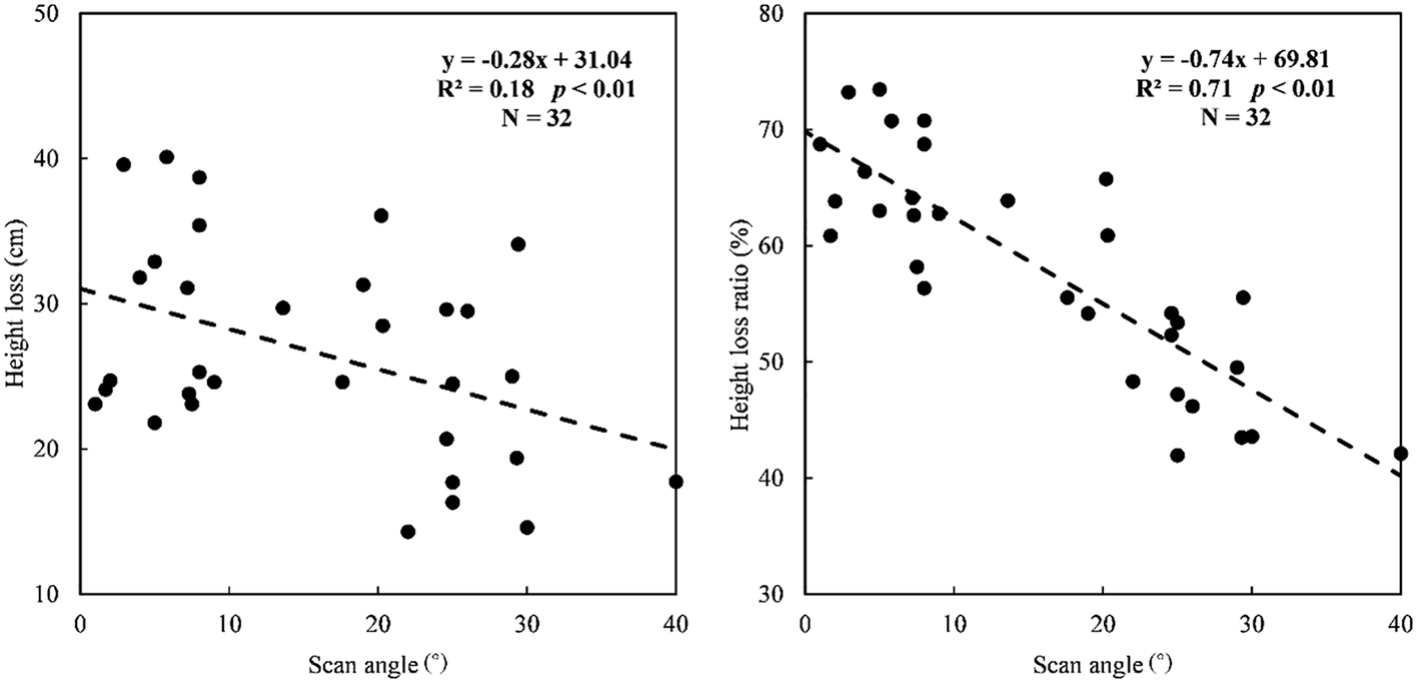
The relationships between scan angle and height loss **(A)** and height loss ratio **(B)**.

The stronger linear negative relationships between scan angle and two height loss indicators were also found when the canopy height ranges of plants themselves were considered (**Figure 4**). The height loss ratio still showed higher correlations with scan angle (R^2^ from 0.78 to 0.86) than height loss (R^2^ from 0.69 to 0.79). The highest correlation between height loss ratio and scan angle appeared in the height range of 25–40 cm (R^2^ = 0.86, *p*< 0.01), while it was 40–50cm for height loss (R^2^ = 0.79, *p*< 0.01). The height loss ratio showed consistent variation with scan angle changes for the height ranges of 25–40cm and 40–50cm (slope = -0.76 and -0.74), which was lower than that in the range of 50–65cm (slope = -0.89). Meanwhile, the maximum fitting values of the loss ratio for three height ranges were different when the scan angle was 0 (66.36%, 70.56% and 76.22%, respectively).

**Figure 4:**
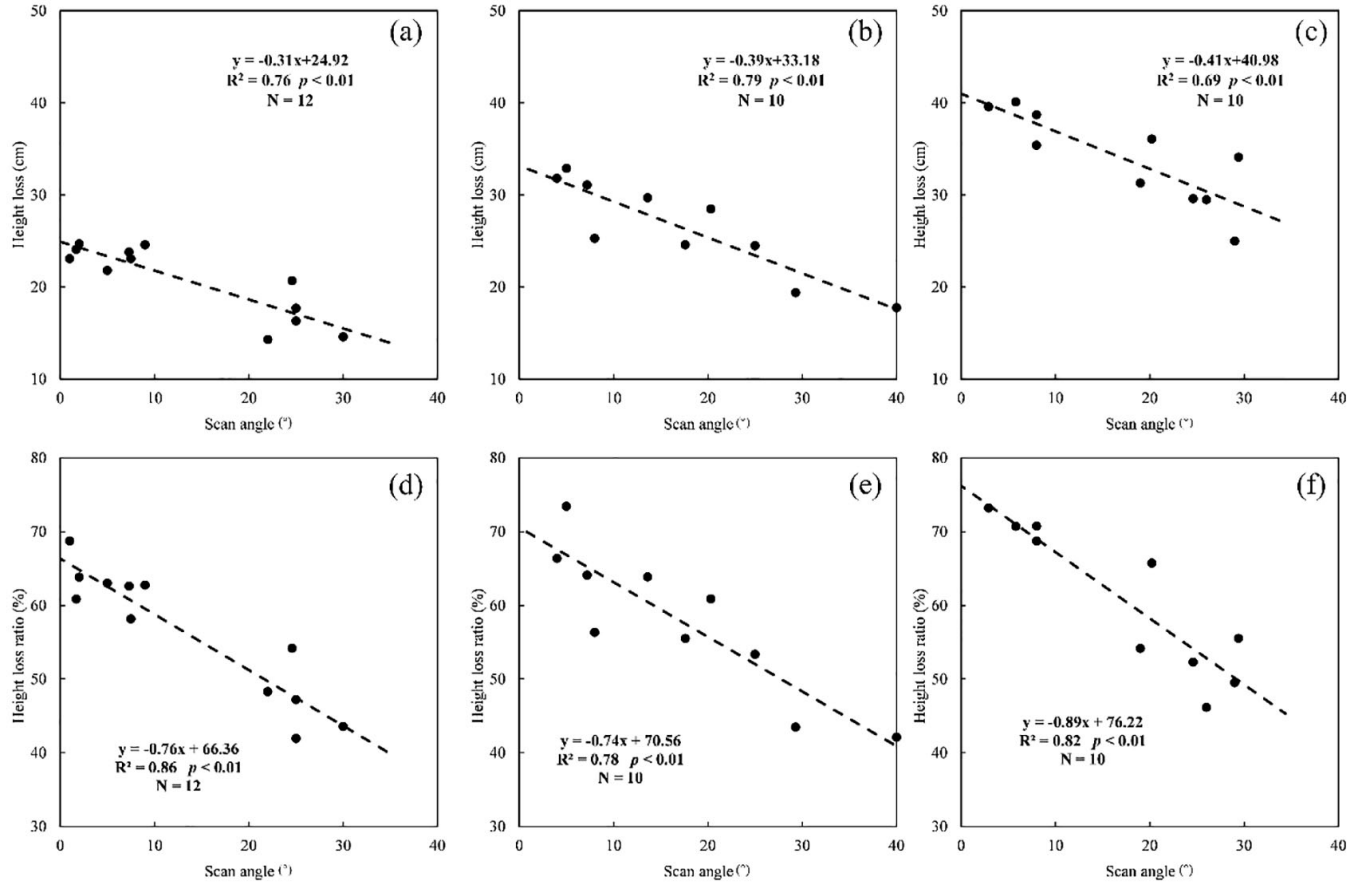
The relationships between scan angle and height loss or height loss ratio at three different height ranges [25–40cm **(A–D)**; 40–50cm **(B–E)**; 50–65cm **(C–F)**].

Grassland canopy height information loss of sample plots showed certain differences in three different height ranges (25–40cm, 40–50cm and 50–65cm) when the scan angle between 0 and 40° (**Figure 5**). With the increase of mean canopy height of plants at plots level, the mean height loss increased from 23.1cm (25–40cm) to 26.8cm (40–50cm) and 34.0cm (50–65cm) and there were significant differences between each pair at 0.01 level. By comparison, the differences of height loss ratio between the three height ranges were non-significant and they showed similar averages with 59.5% in 25–40cm and 57.9% in 40–50cm and 60.7% in 50–65cm, which closed to the average of all samples (58.3%).

**Figure 5:**
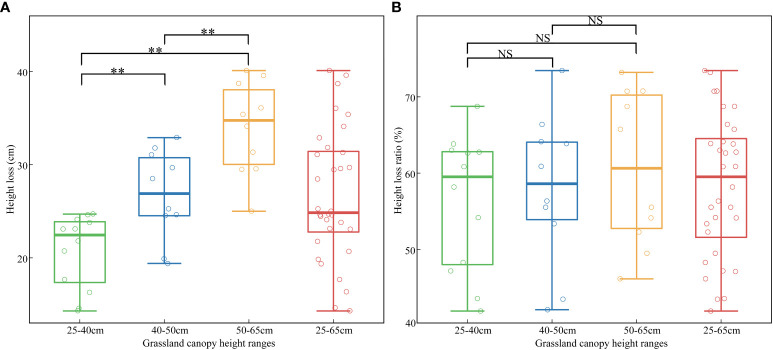
Mean canopy height information loss **(A)** and height loss ratio **(B)** differences of sample plots in three different height ranges (25–40cm, 40–50cm and 50–65cm). ** represents the significance at the level of 0.01 and NS represents non-significant for t-test.

### 3.3 Grassland height after correction

After correcting the loss of grassland height, for the validation dataset, the estimated grassland height based on UAV LiDAR showed significantly improved accuracy with R^2^ increased from 0.23 to 0.68 (holistic correction) and 0.82 (segmented correction), RMSE decreased from 19.17cm to 5.26cm and 3.85cm, respectively (**Figure 6**). It was also demonstrated that the segmented correction performed better than holistic correction for correcting the height loss as shown in **Figures 6B, C**.

**Figure 6:**
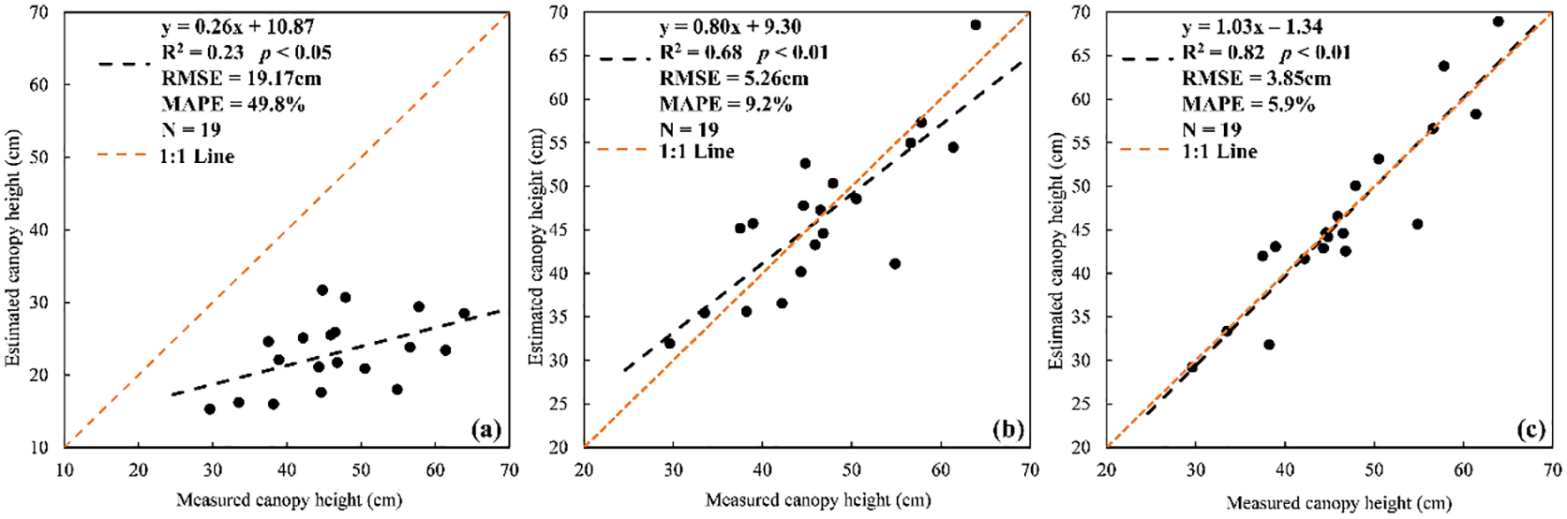
Estimated canopy height versus measured canopy height before **(A)** and after correcting the height loss based on the holistic correction **(B)** and segmented correction **(C)** for validation dataset.

## 4 Discussion

### 4.1 Effects of scan angle

Our study demonstrated that the loss of grassland height was strongly related to the scan angle, and there was a larger height loss with the decreasing scan angle. Actually, with the increase of scan angle, more tilted laser beams hit the targets and there is a larger contact surface between the plant individual and the laser beams as well as more chances to encounter gaps within the canopy (Kamoske et al., 2019). It demonstrated that a higher probability of getting information at the top of the plant and penetrating deeper into the dense canopy to obtain the ground when the scan angle is larger. Moreover, we compared the estimated height of plant and ground, then found limited differences for ground elevation and larger differences for grassland canopy height between two scanning with various scanning angles, which indicated the height loss came from the underdetection of highest points of plant canopies. Although height information loss was common in grassland when estimating height based on UAV LiDAR, whether it used point-based, CHM-based or voxel-based methods (Streutker and Glenn, 2006; Guo et al., 2021b), this part of the loss could be effectively corrected by scan angle according to our study. After the correction based on scan angle, the accuracy of grassland canopy height estimation based on UAV LiDAR was significantly improved (**Figure 6**) and closer to the real. These enabled further accurate estimation of ecological parameters related to grassland canopy height, such as grassland aboveground biomass (Michez et al., 2019; Morais et al., 2021), functional diversity (Lavorel et al., 2007) and revegetation effectiveness (Li et al., 2019).

Moreover, simple linear relationships between scan angle and the loss of grassland height were built based on the assumption that all the height information loss was caused by scan angle in our study. However, obtaining the peak information of each individual plant by UAV LiDAR in grassland was still a challenge due to the small size of individual grassland plants or the lack of obvious crowns (Zhao et al., 2022). Thus, determining the amount of height information loss caused by scan angle through multiple repetitive observations on the same grassland as well as combined with the results of model simulations would be conducive to extracting grassland canopy height information or other structural traits more accurately based on UAV LiDAR. Multiple scanning observations can help us to determine the height loss caused by scanning angle and can also be applied in the grassland height estimation by averaging the heights of multiple corrections.

Specially, it was also informed that the loss of grassland height caused by scan angle was further related to the height of plants themselves. Higher grassland plants tend to have more height loss within the same range of scan angles, while the mean height loss ratio was similar among three height ranges (**Figure 5**), which could explain the lower correlation between scan angle and height loss than height loss ratio without the height separation process (**Figure 3**). Although the use of height loss ratio reduced the effects, we still obtained a better performance of height estimation after correcting the loss of grassland height based on segmented correction than holistic correction (R^2^ = 0.68 and 0.82, respectively) because of the better fitting relationships between scan angle and height loss ratio in three height ranges (25–40cm: R^2^ = 0.86, 40–50cm: R^2^ = 0.78 and 50–65cm: R^2^ = 0.82) than that in the whole height range (25–65cm: R^2^ = 0.71). Therefore, the application of the correction method would depend on the grassland condition. The holistic correction method could be established and used to correct the loss for the grassland with small height differences in a regional scale. But for the grassland with various layers of height, there would be a better performance using the segmented correction method, which needed a step about layering the grassland heights in advance. A few metrics relative to the grassland height might be considered for the preprocessing of layering, which could be predicted accurately by remote sensing, such as biomass and some biochemical traits (Jin et al., 2013; Lussem et al., 2019).

Additionally, the performance of estimated grassland height was inconsistent between three height ranges (**Figure 2**), which mainly depended on the effects of various scanning angles. Within each height range, scanning angles determined the loss of grassland height and further affected the height estimation accuracy. For example, the negative relationship between the field-measured and estimated canopy height was found in the height range of 40-50cm, because the sample plot with relatively higher plants (>45 cm) had smaller scanning angles and resulted in larger height loss.

Although our results demonstrated that the height loss correction models were effective when the scanning angle was less than 40 degrees, whether there was still a significant linear relationship between scanning angle and height loss or would tend to saturate when the scanning angle larger than 40 degrees needs to be further explored. Limited by the parameters of the UAV, the scanning angle was difficult to obtain the complete data between 0-90 degrees, it could be considered in combination with the terrestrial laser scanner or simulation to complete the entire range of data acquisition and analysis in the future study.

### 4.2 Height estimation among different grasslands

Our results demonstrated that the ability of UAV LiDAR for grassland canopy height estimation varies across different ranges of grassland height (**Figure 2**). Different grassland types commonly showed various structures due to the differences in dominant species and the height of the grassland was one of the main manifestations, which was even used to distinguish the grassland types (Wu et al., 2017). Moreover, grassland types or conditions were usually considered to be an important input or influence factors to estimate ecological parameters by remote sensing (Li et al., 2016; Guo et al., 2021a; Gholizadeh et al., 2022a), which were also considered to be a vital cause of the different performance of UAV LiDAR-derived grassland height.

In this study, we proved that grassland height estimated by UAV LiDAR data was severely affected by scan angle and it could be estimated with high accuracy after the scan angle-based correction on the temperate meadow grassland. By comparing the profiles of grassland height estimated by UAV LiDAR data along the direction perpendicular to flight path (**Figure 7**), the estimated height showed inverted parabolic trends with scan angle both in our study and in the temperate typical steppe located in Inner Mongolia grassland ecosystem research station (43°38′ N, 116°42′ E). The similar performance in two temperature grassland types demonstrated the universal effects of scan angle on grassland canopy height estimation based on UAV LiDAR.

**Figure 7:**
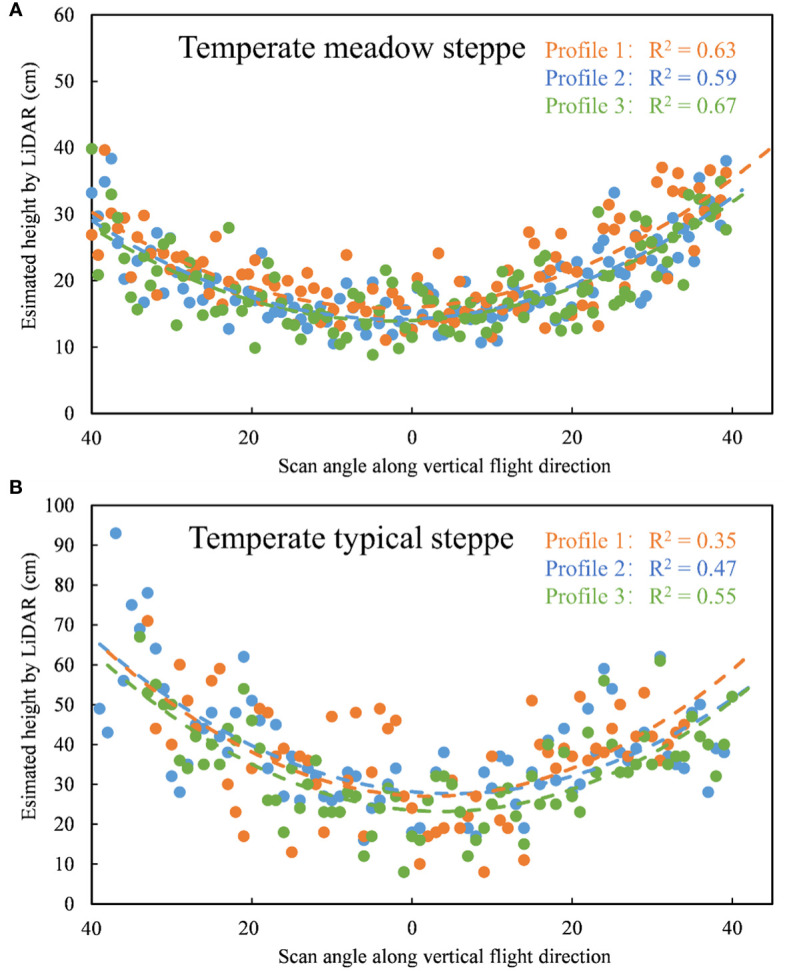
Profiles of estimated grassland height with scan angle along the direction perpendicular to the flight path in our study **(A)**. temperate meadow steppe) and Inner Mongolia grassland ecosystem research station **(B)**. temperate typical steppe).

However, we did not determine the specific relationships between scan angle and the loss of grassland height and analyze the difference in the relationships between the two grassland types due to the lack of ground samples data. Conducting similar work in various grassland types to validate the universality of the method would be valuable for building a more robust grassland height estimation model under multiple conditions. Compared with the meadow steppes, the lower vegetation cover and simpler vertical structure of typical or desert steppes may affect the height correction results at different height layers due to the differences in the reception of UAV LiDAR signals. For typical or desert steppes, the methods usually were conducted by constructing empirical relationships between *in situ* data and optical remote sensing data (Lussem et al., 2019),or building complex machine learning models, which required large amounts of ground survey data(Viljanen et al., 2018; César De Sá et al., 2022). Therefore, analyzing the performance of estimating the height towards different grassland types combining LiDAR and optical data is a necessary future work for ecological applications.

### 4.3 Impacts of UAV LiDAR data for grassland height estimation

We used a CHM-based method that relied on the UAV LiDAR data to estimate the grassland height, and the spatial resolution of CHM was commonly regarded as a crucial issue affecting the accuracy of height estimation. The selection of optimal spatial resolution of CHM had been widely explored in forest ecosystems, while it was rarely reported in grasslands. For example, it was proved that the spatial resolution of CHM between 0.1m and 2m all performed well in estimating the canopy height of forests (Yin and Wang, 2019; Gu et al., 2020; Picos et al., 2020). However, we found that grassland height estimation showed less tolerance to the CHM resolution than forests after testing the performance of CHM with varied spatial resolutions (0.05m, 0.1m, 0.2m, 0.5m and 1m). We demonstrated that it had the best performance at the spatial resolution of 0.1m and it might fail when the spatial resolution of CHM is coarser than 1m (*p* > 0.05) before correction (**Figure 8**), which might be relative to the smaller size of grassland plants. The coarser spatial resolution would lose the details of grassland individual canopy, while finer spatial resolution might introduce redundant information (i.e., canopy gaps) (Sadeghi et al., 2016). The best resolution should be appropriate to or better than the size of the individual plants. Moreover, the effect of CHM spatial resolution on canopy height estimation varies across forest types (Sadeghi et al., 2016; Yin and Wang, 2019; Liu et al., 2020) due to various species, sizes and postures of plants. The optimal spatial resolution of CHM for estimating grassland canopy height could be a focus of future work and it might also be inconsistent for different grassland types.Additionally, although the accuracy of grassland canopy height estimation was affected by the spatial resolution of CHM, the performance in each spatial resolution was improved after correcting the effects of scan angle. The scan angles of adjacent points were basically the same and the angle differences along the direction perpendicular to the flight courses still existed. Therefore, the effects of scan angle were relatively independent of the spatial resolution of CHM.

**Figure 8:**
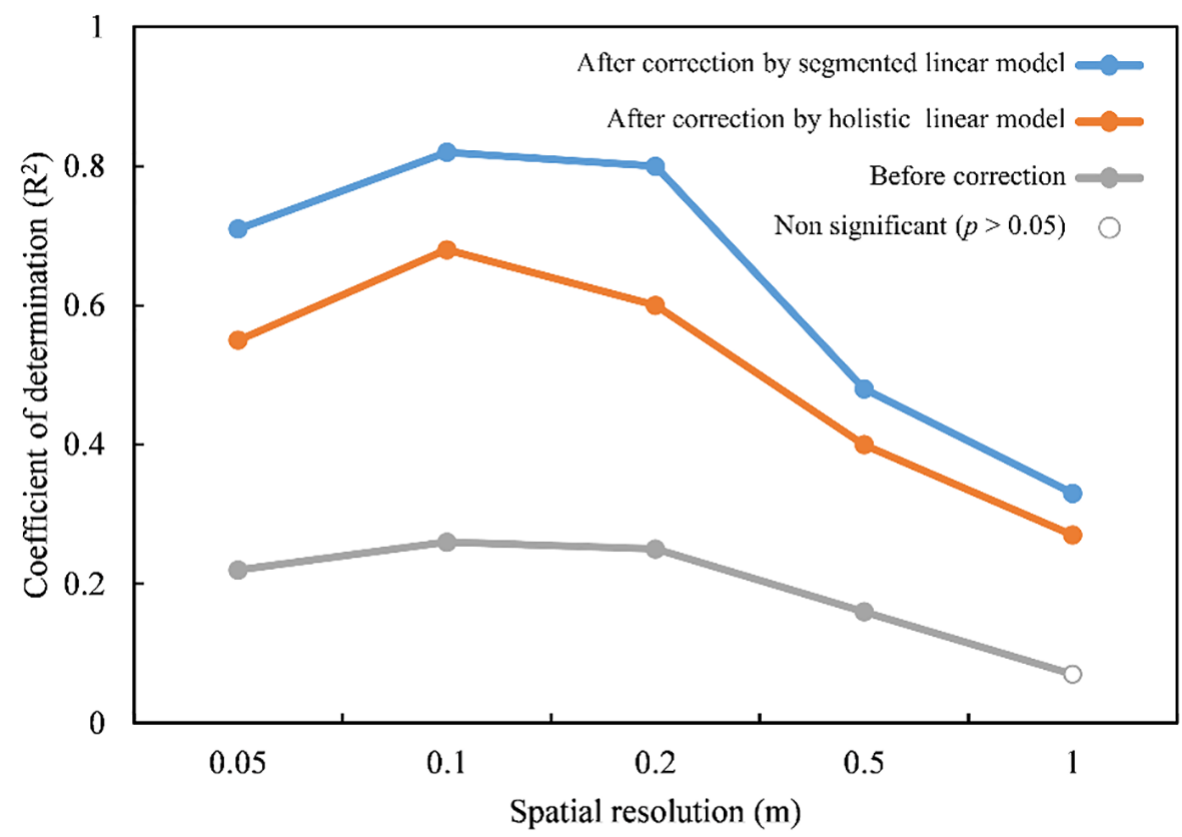
The performance of the estimated grassland height based on UAV LiDAR-derived CHM with varied spatial resolutions.

Point cloud density was considered to be another factor from UAV LiDAR data affecting the accuracy of grassland canopy height estimation (Peng et al., 2021; Zhao et al., 2022). Lower point cloud density usually decreases the probability of obtaining information about the targeted plants. For example, we estimated grassland height at the spatial resolution of 1×1m including 10×10 pixels, but the estimated maximum height from UAV LiDAR data did not occur at the same location as the maximum height from the ground sample survey data shown in several sample plots both before and after correcting the loss of height. The point densities of these sample plots were counted and found to be lower (124–164 points/m^2^) than the average point cloud density (248 points/m^2^). Although it did not significantly affect the estimation of mean height at the sample plot scale in our study, it could lead to that the highest plant individuals were not always detected at the top position and this relative information was lost due to the insufficient point cloud density. Moreover, it had been shown that the accuracy of monitoring grassland height tended to stabilize when the point cloud density exceeded a certain threshold for terrestrial laser scanning (Zhao et al., 2022), but there were fewer studies quantified this relationship based on UAV LiDAR-based height and the point density threshold. Usually, points cloud density is related to the UAV or sensor parameters (i.e., flight area, flight speed and pulse frequency). Therefore, reconciling appropriate point cloud density and projected grassland monitoring area would be clearly an important issue in mapping structural traits related to canopy height over regional grasslands.

## 5 Conclusions

In this study, we demonstrated that scan angle was a main cause for the difference of grassland canopy height loss in the horizontal direction and developed a grassland canopy height correction method based on scan angle. There were significant linear relationships between height loss indicators and scan angle, and height loss ratio had a stronger relationship with scan angle than height loss, which was used to correct the height loss of grassland canopy estimation. After correction, the accuracy of grassland canopy height estimation was improved with R^2^ from 0.23 (RMSE = 19.17cm) to 0.68 (RMSE = 5.26cm) for holistic correction and 0.82 (RMSE = 3.85cm) for segmented correction. Moreover, due to the influence of the canopy height of the grassland itself, different fitting linear relationships between scan angle and height loss indicators among three height ranges were found, which explained the better performance of segmented correction method. By exploring the effects of scan angle on grassland canopy height estimation, our study demonstrated the necessity for correcting the effects of scan angle on LiDAR-derived canopy height in grasslands, which should be a crucial preprocessing step for estimating grassland canopy height accurately.

Our study illustrated that the grassland canopy height can be estimated with high accuracy by UAV LiDAR data after correction based on scan angle. Continuous height maps for regional grassland can be described and further used for ecological applications, such as grassland aboveground biomass estimation or functional diversity assessment. Currently, research about the height estimation in grassland by UAV LiDAR has been increasing, we suggest some crucial issues caused by grassland conditions, data acquisition and environment factors should be addressed in the future for improving the ability of monitoring grassland structure and functions. These will provide references to bridge the scale gap of grassland structural trais estimation between sample plot measurements, regional UAV or airborne and even global satellite monitoring.

## Data availability statement

The raw data supporting the conclusions of this article will be made available by the authors, without undue reservation.

## Author contributions

All authors listed have made a substantial, direct, and intellectual contribution to the work and approved it for publication.
